# Self-Healing Polymer Nanocomposite Materials by Joule Effect

**DOI:** 10.3390/polym13040649

**Published:** 2021-02-22

**Authors:** Jaime Orellana, Ignacio Moreno-Villoslada, Ranjita K. Bose, Francesco Picchioni, Mario E. Flores, Rodrigo Araya-Hermosilla

**Affiliations:** 1 Magíster en Química con Mención en Tecnología de los Materiales, Universidad Tecnológica Metropolitana, Santiago 7800003, Chile; jaime.orellanao@utem.cl; 2 Programa Institucional de Fomento a la Investigación, Desarrollo e Innovación (PIDi), Universidad Tecnológica Metropolitana, Ignacio Valdivieso 2409, P.O. Box 8940577, San Joaquín, Santiago 8940000, Chile; 3 Laboratorio de Polímeros, Instituto de Ciencias Químicas, Facultad de Ciencias, Universidad Austral de Chile, Valdivia 5090000, Chile; imorenovilloslada@uach.cl; 4 Department of Chemical Product Engineering, ENTEG, University of Groningen, Nijenborgh 4, 9747AG Groningen, The Netherlands; r.k.bose@rug.nl (R.K.B.); f.picchioni@rug.nl (F.P.)

**Keywords:** self-healing, functional polymers, conductive nanofillers, nanocomposites, Joule effect

## Abstract

Nowadays, the self-healing approach in materials science mainly relies on functionalized polymers used as matrices in nanocomposites. Through different physicochemical pathways and stimuli, these materials can undergo self-repairing mechanisms that represent a great advantage to prolonging materials service-life, thus avoiding early disposal. Particularly, the use of the Joule effect as an external stimulus for self-healing in conductive nanocomposites is under-reported in the literature. However, it is of particular importance because it incorporates nanofillers with tunable features thus producing multifunctional materials. The aim of this review is the comprehensive analysis of conductive polymer nanocomposites presenting reversible dynamic bonds and their energetical activation to perform self-healing through the Joule effect.

## 1. Introduction

Self-healing is the natural ability of living organisms to repair tissue damage and to endure harsh environments through dynamic mechanisms [1,2]. Inspired by nature, self-healing materials are typically designed with synthetic polymeric components that undergo self-repairing mechanisms under different stimuli conditions [2]. Polymeric components can go through the self-healing process aided by grafted functional chemical groups on the backbone of the polymer [3,4,5]. Such functionalized polymers bearing chemical groups that display reversible bonds represent a great advantage in terms of physical and chemical responses to different stimuli for self-healing. Among several factors, the tunable melting point and melt flow in functional polymers are useful parameters to design materials able to undergo crack healing processes. The latter has been demonstrated to be a key factor for repairing structural damage [3], shape recovery [6], and dimension stability of materials [7,8,9].

The search for highly efficient self-healing polymers and nanocomposites has been addressed through different approaches [10]. Particularly, polymer matrices used in nanocomposites ranged from rubbers to thermoplastics/thermoset polymers [11,12,13]. Regarding thermoset and crosslinked rubber matrices, there are many issues to overcome, mainly due to their lack of re-processability after service, as compared thermoplastics. However, by combining specific functionalities in the nanocomposite, such as reversible polymer networks and active nanofillers, many possibilities for producing self-healing nanocomposite materials finely tuned at the nano scale have been opened [4].

The key characteristic of these chemically functionalized nanocomposite materials is the production of interactions that respond to different stimuli, such as heat, light, or electricity, to perform self-healing [14]. In addition, self-healing polymers and nanocomposites appear to be profitable and promising alternatives for producing long-lasting materials [15,16]. This stems from the fact that nano/composites are widely used in applications such as the automotive industry [17], textile industry [18], electronics [19], to name a few examples. Therefore, self-healing composites represent a great alternative to overcome environmental issues generated by thermoplastics and thermoset land-fields, so having more durable and eco-friendly materials is a current challenge that the academy and R&D industrial departments have decided to tackle [20,21].

Functional polymers and fillers represent a great advantage for producing self-healing nanocomposites. This comes from their high amount of production as commodities and the endless possibilities of combination between them to generate composites that show different characteristics and applications. Self-healing polymer nanocomposite systems have gained a lot of attention due to the combination of functional polymers with different types of nanofillers such as silica, clay, metal, and carbonaceous nanoparticles. These nanofillers substantially improve the strength, modulus, and toughness of polymeric matrices, as well as the formation of the percolative network to transport external stimuli inside the polymer matrix for repairing [22].

For instance, electrically self-healing nanocomposites work through nanoscopic heat generation when an electric current passing through a conductive nanostructured network (e.g., well-connected CNTs, metallic nanoparticles, graphite/graphene networks). The so-called Joule-effect (or resistive heating) activates the thermal self-healing ability of self-mendable matrices to heal damage on local areas [23,24].

To fulfill the condition of self-healing, two main approaches have been extensively reported in the literature: the so-called extrinsic and intrinsic self-healing mechanisms. The extrinsic one is based on micro-capsular and micro-vascular systems that contain repairing agents [25,26]. These agents generally polymerize, repairing the damage [27,28]. The problem lies in the limited amount of repair agent [9], where upon its depletion, the material loses the ability to self-repair [14]. Intrinsic self-repairing systems have reactive groups bearing polymer backbones [3] that undergo reversible bond interactions, both covalent and non-covalent, upon external stimuli. These intrinsic systems can theoretically be repaired many times due to their intrinsic character [14].

**Figure 1:**
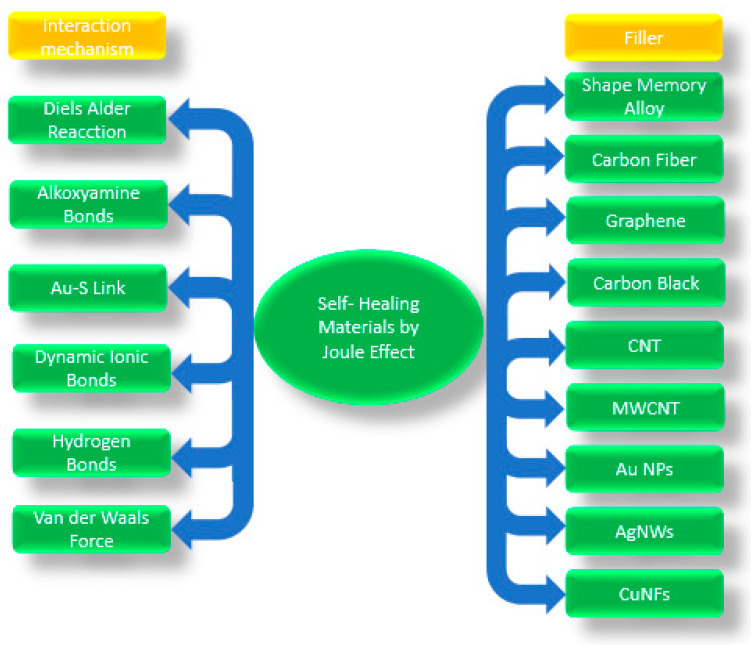
Schematic representation of self-healing material composition [29].

Self-healing materials can present many bond interactions, enclosed in two large groups: the so-called non-covalent and covalent interactions (Figure 1). The former includes lower energy dynamic non-covalent bonds such as van der Waals interactions, π–π stacking, dipole–dipole interactions, hydrogen bonding, ionic interactions, metal–ligand coordination, and host–guest interactions [28]. The latter includes the highest energetic group, dynamic covalent bonds. Although in this group there are many mechanisms, the most commons are Diels-Alder chemical interactions, transesterification reaction, disulfide bonds, imine bonds, boron-based bonds, and alkoxyamine [28]. From this group, the Diels-Alder (DA) reaction is highlighted for its thermally self-healing behavior [29].

To induce self-healing processes in polymer nanocomposites, polymer chains may diffuse into the damaged zone. For polymers, mobility of macromolecular chains occurs at temperatures above their glass transition [28], so that temperature plays an important role in polymer self-repairing [30]. It can be provided by thermal energy such as conventional heating in ovens [29], or by heating by microwaves and infrared irradiation [31,32,33,34]. Additionally, inductive heating can be applied by using current coils located in the damage region to be repaired [35,36,37]. Finally, Joule heating occurs in conductive composite materials, mainly aided by the conductive filler network when a current circulates through it, as previously mentioned [37,38].

Currently, the production of high-tech manufactured materials requires both damage detection and self-repair to avoid waste [39,40], so the concept of in-service repair is gaining strength, especially in materials that are difficult to access [36]. Therefore, in this review we explore the Joule effect as an ideal candidate as a self-healing stimulus for in-service self-healing.

**Figure 2:**
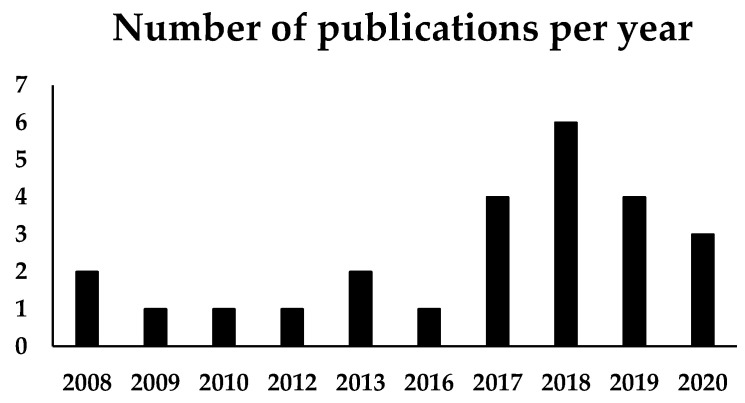
Number of publications related to self-healing materials by Joule effect.

This review briefly covers the general topics of self-healing nanocomposites [41] and further focuses on healing by Joule effect found in literature (Figure 2). The approach of using Joule heating in conductive smart materials covers functional polymer matrices in combination with conductive nanofillers. We identify the chemical pathways that are thermally stimulated in which dynamic covalent bonds, such as the Diels-Alder interactions, alkoxyamine bond and Au-S bonds, are present. We also identify dynamic ionic bonds, in which butyl bromide-based molecules are found, and we also find that supramolecular interactions with the so-called thermoplastic/thermoset blend self-healing system are activated by Joule effect.

## 2. The Joule Effect in Materials

The Joule effect corresponds to the generation of heat when an electric current passes through a conductive material. This heating phenomenon was described in 1841 by James Joule. It occurs when electrons collide with the atomic lattice of conductive materials, ending in the transference of its kinetic energy in the form of dissipated heat. It is also called resistive heating after the model of Paul Drude in 1900 [42]. This phenomenon is widely used for welding metals or surgical operations without bleeding, to name a few examples. Two important factors must be met for the Joule effect to be fulfilled: the material must be both electrical and thermally conductive. This effect can be used to heal a material externally and the power needed to activate, for instance, a self-healing material depends on the intensity to achieve the required activation energy [43]. The effect is usually observed in materials formulated with macro fillers such as fibers, wires, and films.

Internal Joule heating can activate the self-healing mechanism in conductive materials. For this, the material must be intrinsically conductive; however, it is widely known that polymers do not conduct electricity. For this reason, nanofillers play an important role in this type of internal Joule heating, due to their large area-to-volume ratio and excellent percolative network properties, which provide both electrical and thermal conduction [44,45,46,47].

**Figure 3:**
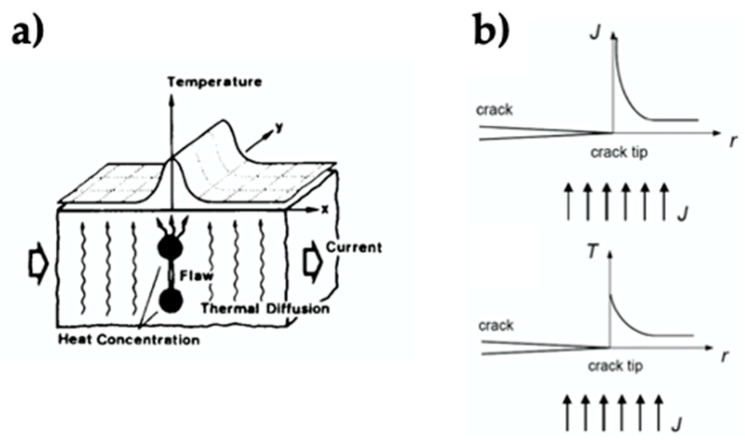
(**a**) Localized temperature rise [48]. Adapted with permission from Sakagami, T; Transactions of the Japan Society of Mechanical Engineers Series A; published by J-Stage, 1992; (**b**) Crack tip fields: electric current density (J) and temperature (T) [49]. Adapted with permission from Liu, T; Engineering Facture Mechanics; published by Elsevier, 2014.

The main characteristic of internal heating is related to the localized increase in thermal energy in microcracks and cracks, raising the temperature of the damaged area in a localized way due to the change of direction of the electric current at the tips of the cracks [48] (Figure 3). When the direction of the electric current is perpendicular to the crack, the temperature at the tip of the crack will be at its maximum; then, the temperature at that region depends on the electric current supplied, the geometry of the crack, and the load mechanics of the material [49] (Figure 3). This phenomenon can be used to perform self-healing functions, since the ideal thermal conditions are found at the tip of the cracks, optimizing the use of energy. This localized temperature, due to the crack, can also be used to detect cracks in the material, since these temperatures can be monitored with infrared cameras [48].

Another interesting application of the Joule effect in self-healing materials is the arrest of crack growth. For instance, microcracks presented in a given material have the tendency to grow at their tips, and in the long term, this effect is very detrimental, eventually producing catastrophic failures. The localized increase in temperature at the tip of the crack is used to generate a localized fusion of the matrix, then the tip cools down and contracts, thus stopping the crack growing [50,51]. This effect can also be used as a fault detector, because if there is a localized increase in temperature, it can be detected by infrared cameras [48,49,52,53].

The Joule effect can be used in self-healing materials as an internal or external source of heating. To observe this effect, the thermal properties of the matrix must be well characterized, since applying a certain range of current will allow fault detection temperatures to be obtained. The Joule effect can be used both in the detection and repair of crack damage, leading to intelligent self-healing materials. Some of its main benefits include the simplicity of application, its low energy cost, and the possibility of applying the heating to the material without the need for intervention, resulting in intelligent action in-service.

## 3. Filler Effect

Fillers give new properties to polymer materials. Carbonaceous fillers such as carbon black, carbon nanotubes (CNTs), carbon nano-onions (CNOs) and graphene provide thermal and electrical conductive properties, as well as good mechanical performance [54,55,56,57,58,59]. Likewise, metallic fillers such as silver nanowires or copper nanofibers provide excellent electrical and thermal conduction and great mechanical performance, as well as being optically transparent [60,61,62,63]. This opens a large number of applications, such as electro-thermal heaters [64,65], strain sensors [66], applications in biomedicine [67] and electrically conductive adhesives [68], to name a few examples. The problem is that the vast majority of polymers are very poor conductors, so the addition of conductive fillers provides conduction when the filler forms a percolative network of both electrons for electrical conduction and phonons for thermal conduction [69]. The correct formation of the percolative network depends on the volume fraction of the filler in the matrix with physical contact between them for electrical conduction [70,71]. Additionally, the polymeric matrix acts as both electrical and thermal barrier. For this to happen, an effective dispersion and stabilization of the filler must be achieved before a given nanocomposite reach percolative pathways [72]. Thermal conduction in amorphous polymers occurs by phonons, but as it is not an ordered structure, vibrations are disordered, and thermal conduction is not highly effective. Adding fillers improves phonon transmission, so interfacial covalent bonds play an important role in the propagation of phonons from matrix to filler [73], but the presence of a large number of bonds also has a negative effect, since the phonons are dissipated by these bonds, and the thermal conductivity decreases [73,74].

## 4. Intrinsic Self-Healing Material by Joule Effect

### 4.1. Dynamic Covalent Bonds

Within the category of dynamic covalent bonds, only a few chemical routes can be classified to produce intrinsic self-healing polymer materials. Among them, the most commonly used is pericyclic addition reactions [75]. The Diels-Alder [4 + 2] cycloaddition reaction is one of the best reversible covalent reactions for the production of self-healable materials. Assuming that the adduct reaction is in a dynamic equilibrium, the crosslinking density and kinetics are influenced by temperature, favoring the endothermal reaction and shifting the reaction to the initial reagents in DA reaction, thus producing a decrease in the crosslink density [76].

#### 4.1.1. Diels-Alder Reaction

Diels-Alder (DA) reaction is a pericyclic addition between a diene and a dienophile that generates a cyclohexene adduct. This reaction is reversible when the temperature increases, and the formed DA adduct is broken into a reaction called retro Diels-Alder (r-DA). When the temperature begins to drop, it allows the cycloaddition reaction to take place again, completing the DA/r-DA cycle that confers the reversibility characteristic to polymers bearing diene and/or dienophile functional groups [14]. DA adducts can be formed between DA-functionalized polymer mixtures, as well as cross-linking agents, or the adducts can also be formed through covalent interfacial bonds between a DA-functionalized polymer matrix and carbonaceous nanofillers [77,78,79]. The latter provides reversibility to a cross-linked polymer nanocomposite network at the nanoscopic interface [80].

The molecules involved in DA and r-DA reactions establish the condition of reversibility in cross-linked polymeric network, favoring the self-healing of a given material by thermal energy. Using this reaction, the curing process is relatively simple to induce at low temperature [81]. The formation of covalent bonds by Diels-Alder reaction occurs in a temperature range from 50 to 80 °C [82]. It should be noted that the components frequently used to form Diels-Alder adducts are furan and maleimide [83], and the breaking of these bonds (r-DA) occurs predominantly between 90 and 150 °C [39,83].

**Figure 4:**
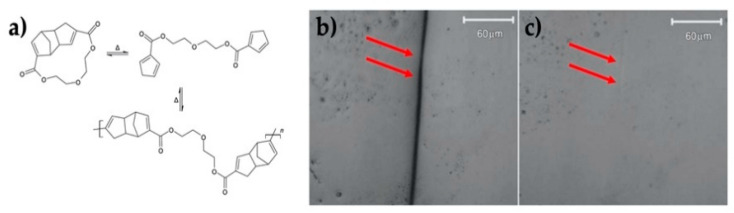
(**a**) Diels-Alder and retro Diels-Alder reactions of mendomer 401, (**b**) microcrack before and (**c**) after microcrack healing by resistive heating [38]. Adapted with permission from Park, J.S; Journal of Composite Materials; published by Sage, 2008.

A series of polymeric materials, where the DA reaction is used to produce reversible covalent bonding for self-healing, is described in the literature [81,84,85,86]. One of the earliest Joule effect-induced self-healing systems was produced by using cyclopentadiene, which can act as both diene and dienophile in DA reaction [81]. Jong Se Park’s group used the dicyclopentadiene, called as mendomer 401, as a self-healing polymer, and coated it onto a graphite fiber/epoxy substrate, which was responsible for generating heat through Joule heating. The material was cured under vacuum at 150 °C for 5 h, and then cracks were induced by folding the material. The external Joule heating occurred, applying an electrical current of 2 A for 20 min, thus inducing the r-DA reaction of the polymer matrix, and the polymer flowed to heal the cracks by Joule heating [38] (Figure 4).

Subsequently, Park’s team manufactured a board composed of layers of carbon fabric and mendomer 401. The composite was cured under vacuum at 150 °C for 20 h, and the material was bent to generate microcracks. The heat was generated by applying an electrical current of 0.5 A, producing a temperature of 150 °C, ideal for the r-DA reaction, allowing the flowing of the de-crosslinked mendomer 401 to heal the material upon cooling [87].

**Figure 5:**
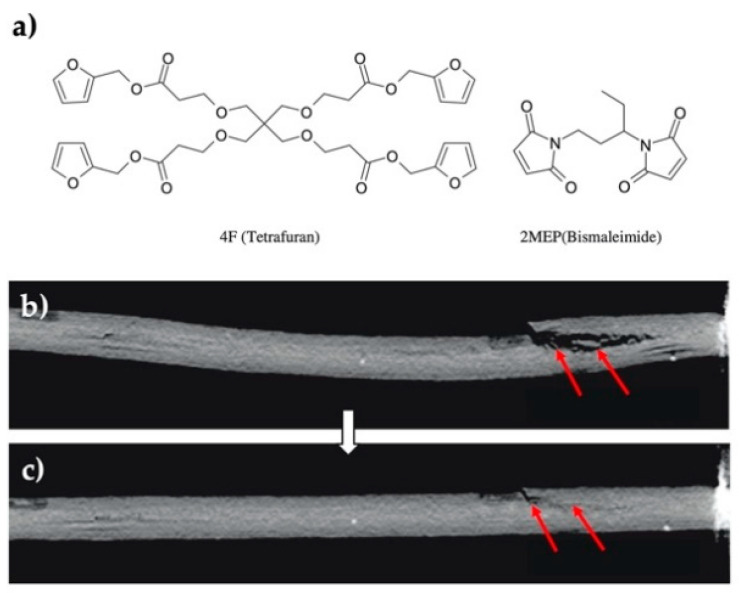
(**a**) Chemical structures of monomers used to make 2MEP4F, (**b**) section views of sample 1 using X-ray tomography before, and (**c**) after self-healing [88]. Adapted with permission from Park, J.S; Composites Science and Technology; published by Elsevier, 2010.

Later, with advances in reversible reactions, Park et. al. also tested materials composed of molecules bearing furan or maleimide functional groups reinforced with one-dimensional carbon fibers as filler for heating through the Joule effect. Specifically, tetrafuran (4F) was used as the diene, and bismaleimide (2MEP) as the dienophile. The material was prepared by injection molding; 4F and 2MEP were heated separately until melting, and then mixed by using a mixing nozzle, and placed into a vacuum mold preloaded with carbon fiber as filler. The formed material was subjected to a current of 1.2 A, which generated a temperature of 110 °C to achieve the r-DA reaction. The composite material displayed Joule heating, shape memory, and a self-healing efficiency of 90% for up to several cycles if the integrity of the fibers was not compromised, and about two cycles with an efficiency of 60% if the fibers were damaged. The drawback of this electrically self-healing material is that if the conductive fibers break, the filler loses its percolative feature, hindering the Joule effect [88] (Figure 5).

In another work, Willocq et al. used a polymer matrix composed of poly (ester-urethane) furfuryl, poly (ε-caprolactone) modified with maleimide groups, and 2 wt.% MWCNT as filler. The blend generates a percolative network in the nanocomposite, producing a three-dimensionally stable cross-linked material, since the polymer matrix reacts with the surface of the MWCNTs. After damage, the material was connected to a current of 25 V to induce self-healing properties. The DA bonds were formed at 50 °C, and the r-DA reaction at 120 °C. This work was the first reported in the field of self-healing nanocomposites using the generation of Joule heating through a percolative network using MWCNTs [89].

**Figure 6:**
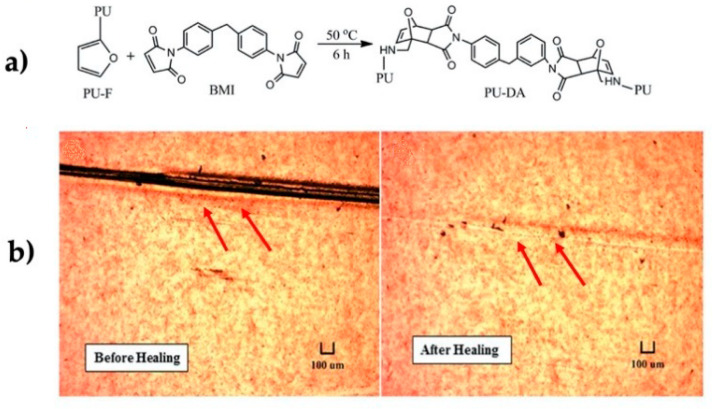
(**a**) Chemical structure of monomers and polymers; (**b**) electrical power induced healing illustrated by optical images of the electrode [90]. Adapted with permission from Tiwari, N; Nanoscale; published by Royal Society of Chemistry, 2017.

In 2017, Tiwari et al. synthesized polyurethane (PU) modified with furfurylamine and cross-linked with 1,1′-(methylenedi-4,1-phenylene)-bismaleimide (BMI), to produce polyurethane with Diels-Alder adducts (PUDA). The functionalized polymer was used to generate a film, which was modified on the surface with (3-aminopropyl) triethoxysilane (APTES). Then, the film was spray coated with silver nanowires (AgNWs), generating a transparent and flexible material with defogging properties, and displaying a percolating network of AgNW. This material presented a sheet resistance of 13.3 ohm/sq. Heat was generated internally applying a current of 12 V for 2 min to reach the temperature where r-DA occurs (120 °C), thus repairing a cut induced on the sheet [90] (Figure 6).

Some of the authors of this review generated conductive nanocomposites that display self-healing properties via Diels-Alder reaction activated by Joule effect. The nanocomposites were prepared by mixing furan-functionalized polyketone matrix, cross-linked with bis-maleimide, and MWCNTs as conductive filler and diene/dienophile groups in DA reaction. Compression-molded bars showed that MWCNTs formed a percolative network with electrical and thermal conduction. DA interactions between matrix and filler allowed the good dispersion and stabilization of MWCNTs inside the matrix. By applying a current of 35 V, the material reached the optimal r-DA temperature of 150 °C, which triggers the self-healing ability on knife formed scratches [79].

**Figure 7:**
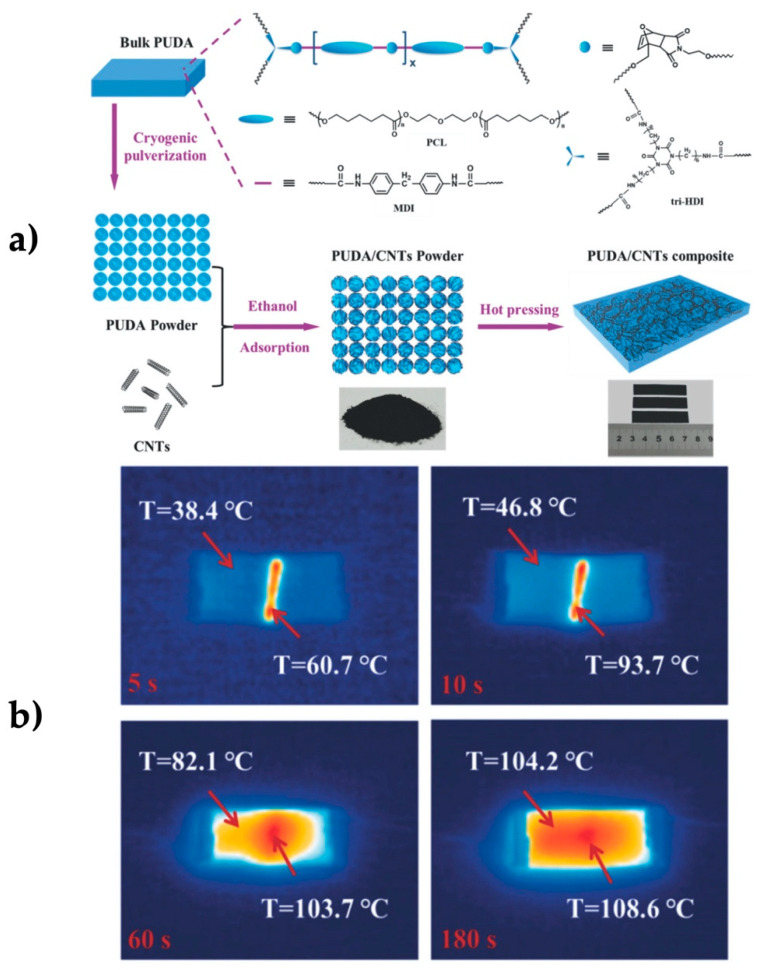
(**a**) Chemical structures of self-healing composite; (**b**) surface temperature of composites connected to electrical current and self-healing [53]. Adapted with permission from Pu, W; Advanced Science; published by Wiley, 2018.

Pu et al. worked on dynamically cross-linked polyurethane compounds bearing Diels-Alder bonds (PUDA) reinforced with CNTs as filler. A prepolymer of polycaprolactone (PCL) and 4,4′-diphenylmethane diisocyanate (MDI), MDI/PCL was synthesized at a ratio of 2:1, heated to 80 °C, then dissolved with trimer hexamethylene diisocyanate (tri-HDI) in anhydrous 1,4-dioxane with a molar ratio of 60:48:8, and polymerized at 80 °C in Teflon molds, obtaining the PUDA. The material was pulverized and the CNTs were dispersed in ethanol, and then the pulverized PUDA was added to the mixture. The mixture was filtered and hot pressed, obtaining the PUDA/CNT. A sample loaded with 1 wt.% of CNTs was cut and the sheet was intentionally turned upside down to emulate internal damage. A voltage of 20 V was applied, which rapidly increased the temperature at the crack region due to the local increase in resistance. The diagnosis of damage on the material after 180 s showed that the entire piece reached 106 °C, indicating a complete cure efficiency of about 98%. The system was also cured with NIR light at 0.4 Wcm^−2^, reaching an efficiency of 97% [53] (Figure 7).

**Figure 8:**
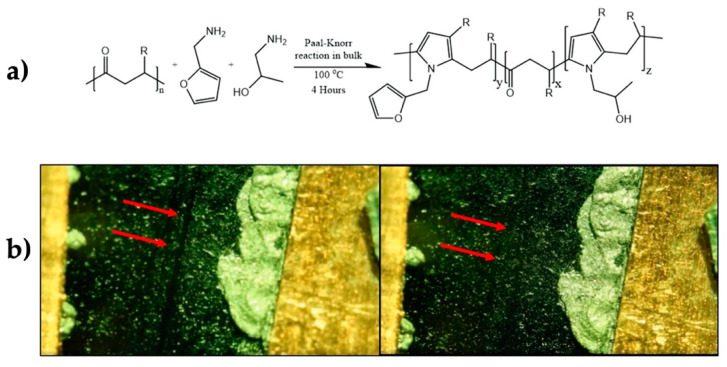
(**a**) Chemical structure of polymer used to prepare composites bearing Diels-Alder functional groups. (**b**) Optical images of self-healing by Joule effect [91]. Adapted with permission from Lima, G.M.R, Polymers; published by MDPI, 2019.

Lima et al. also worked with furan-functionalized polyketones, but containing furfurylamine and grafted hydroxyl groups in the same polymer chain as hydrogen donors. The polymers were cross-linked with different amounts of bis-maleimide and MWCNTs as filler. By changing the ratio between furan and hydroxyl groups, it was possible to tune the crosslinking density and thermomechanical behavior of the material. By compression molding, nanocomposites displaying a percolative network of MWCNTs were obtained. The material was subjected to electric current ranging from 25 to 50 V to reach temperatures from 120 to 150 °C through the Joule effect. The latter allowed the material to self-heal a knife made scratches after achieving 150 °C that triggered the r-DA. The latter promoted the de-crosslinking process (r-DA) of the matrix and its flowing to fill damage regions. Upon cooling, the material re-cross-linked to recover mechanical and conductive properties (conductivity around 1 × 10^4^·S/m) [91] (Figure 8).

#### 4.1.2. Alkoxyamine Bonds

The alkoxyamine bond is an alcohol bonded to a secondary amino group (>N-O-). This bond breaks down homolytically because these types of bonds are thermally labile, forming a nitroxide and a carbon-centered free radical that can react with themselves or other compounds [92]. This bond is thermally activated in a single step, contrarily to DA which requires two steps [93], that makes it interesting for self-healing materials.

Fan et al. perform a design test using a copolymer of polyurethane possessing styrene butadiene blocks modified with alkoxyamine (C-O-N), which act as a shape memory polymer. The copolymer was used to prepare a material including MWCNTs as conductive filler. The system is stimulated by internal Joule heating at 100 °C for 24 h, achieving shape memory recovery of 73.3%. The alkoxyamine groups undergo homolysis reactions when heating, enabling self-healing of the material due to the effect of shape memory of polymer via intrinsic self-healing reversible covalent bonds [94].

#### 4.1.3. Au-S

**Figure 9:**
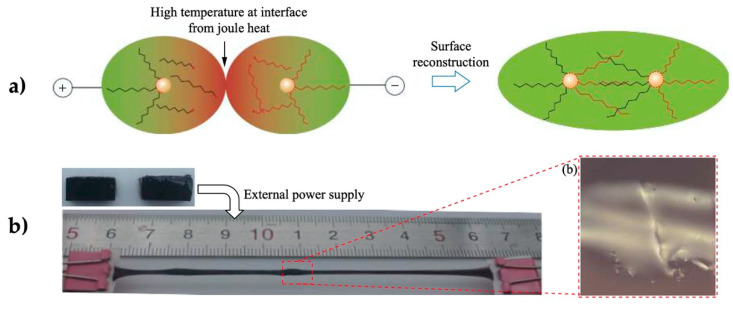
(**a**) Illustration of self-healing process in conductive hydrogel; (**b**) Self-healed material after apply an external current [97]. Reproduced with permission from Wu, B.S; Acta Polymerica Sinica; published by Science Press, 2019.

The Au-S exchange bond is a reversible interaction explored in hydrogels through its thiolate/disulfide exchange mechanism. This bond can be activated in different ways including the Joule effect [95,96]. Wu at al. produced a conductive nanocomposite hydrogel by using gold nanoparticles (AuNPs) coated with N,N-bis(acryloyl)-cystamine (BACA) polymerized in the presence of the semiconductive poly (o-phenylenediamine) and N-isopropyl acrylamide. The hydrogel displayed a Young’s modulus up to 12 MPa and stretching capacity of 2400%. Due to their conductive properties, this hydrogel is self-healed by supplying an electric current of 0.05 A for 15 min, reaching a healing efficiency of 90%. The dynamic thermal instability presented by the thiolate-gold bond (S-Au) allows the self-repairing process [97] (Figure 9).

### 4.2. Dynamic Ionic Bonds

Butyl bromide-based polymers, modified with ionic groups such as imidazole, display self-healing properties due to the dynamic ionic reorganization process [98,99,100]. These polymers can self-heal at room temperature and due to other types of stimuli. Systems based on this chemical pathway can undergo the self-healing mechanism activated by the Joule effect. Le et al. prepared a nanocomposite material combining bromobutyl rubber bearing imidazole groups and CNT as filler by compression molding. Although the material showed self-healing properties at room temperature, after applying an electric current of 15 V, the material reached a temperature of 100 °C, thus accelerating the self-healing effect by the Joule effect [101].

**Figure 10:**
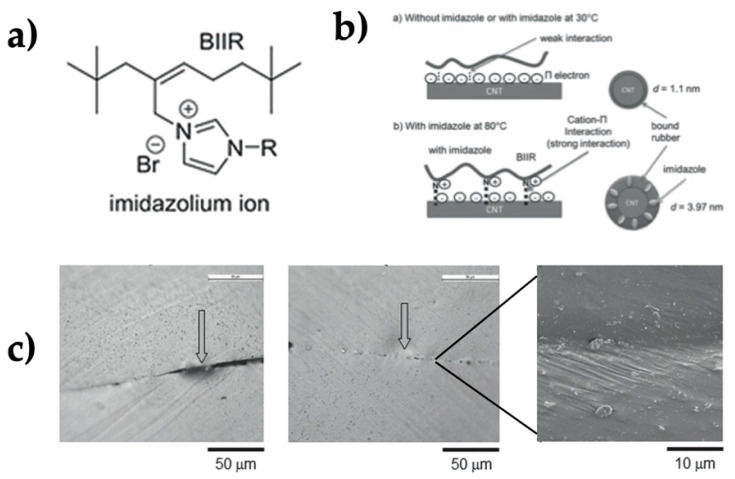
(**a**) Chemical structure of butyl bromide polymer bearing imidazole groups, (**b**) interactions between BIIR and CNTs, and (**c**) SEM images of self-healing after cut the surface [102]. Adapted with permission from Le,H.H; Macromolecular Materials and Engineering; published by Wiley, 2017.

Another interesting material is formed by butyl bromide gum base polymer modified with imidazole groups combined with CNTs. This system has an ionic self-healing mechanism, showing at 80 °C an adequate polymer flowing for self-healing. Specifically, the imidazole group ionizes and interacts with CNTs via cation–π interactions, improving the dispersion of CNTs and thus the mechanical performance. The homogeneous dispersion of CNTs into the composites generates the healing process in the material after applying an electrical current. A system containing 5 wt.% of CNTs as filler displayed a resistivity of 6 × 10^2^ ohm/cm. The temperature reached ranged from 110 to 200 °C when applying a voltage of 38 V for self-healing [102] (Figure 10).

**Figure 11:**
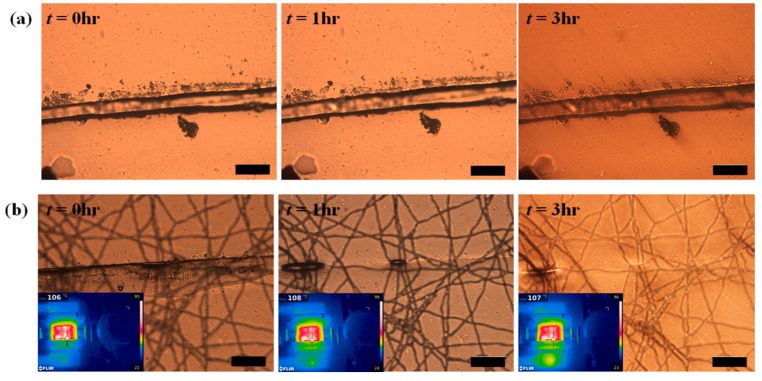
Optical images of the second crack on (**a**) the pristine plane substrate and (**b**) on the substrate heated with the CuNF heater. Scale bars are 100 mm [62]. Reproduced with permission from Lee, M.W; Applied Physics Letters; published by American Institute of Physics, 2017.

Lee et al. also used bromobutyl polymers (rubber BIIR) with a source of heat provided by the Joule effect through sheets of copper nanofibers embedded in polyethylene. The internal heat generation was promoted by the application of a current of 1.18 A and a voltage of 1.3 V. This generated a temperature of 100 °C, thus allowing the mobility of the polymer chains upon physical de-crosslinking to achieve the self-healing of the material [62] (Figure 11).

Kim et al. used bromobutyl rubber BIIR mixed with MWCNTs as filler (10 wt.%) to prepare films by solvent casting. The system was designed to protect underwater surfaces. The self-healing properties were tested under both fresh and sea water. BIIR is a hydrophobic polymer that repairs underwater. For self-healing, it was necessary to apply a voltage of 28.5 V to generate a temperature of 150 °C, which was enough to induce self-healing in the material by ionic interdiffusion [103].

### 4.3. Supramolecular Interactions

Self-healing materials can also be designed using polymers bearing functional groups displaying supramolecular interactions. The latter confer to the material low melting points, useful for polymer chain mobility during the healing process. For instance, an extrinsic self-healing polymer composite material might include capsules filled with a thermoplastic polymer showing a lower melting point than the matrix. During a damage event, heating procedures can melt these low-melting point polymers, allowing them to flow into the cracks, thus filling the damaged region for healing through supramolecular interactions [13,104].

Wang et al. mixed silicone elastomer (Sylgard 184^®^) with a melting glue (Sellery 96-802^®^ glue stick based on ethylene vinyl acetate, EVA) in a 10:1 ratio, and used it to cover a NiTi spring that acted as a Joule heater, forming a shape memory composite material. Once the material was fractured, an electrical potential of 6 V was applied from a battery in tapping mode, thus increasing the temperature of the system by Joule effect, which activated the shape memory effect and the low melting point effect of the silicone elastomer for self-healing [105].

**Figure 12:**
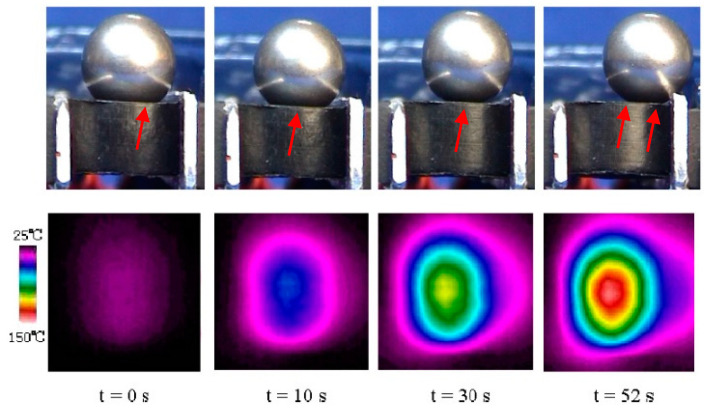
Image sequence, upon Joule heating, of the shape recovery and temperature distribution for S77%/MG15%/CB 8% [106]. Reproduced with permission from Cui, H.P; Smart Materials and Structures; published by IOP Publishing, 2013.

Cui et al. also mixed silicone elastomer (S, Sylgard 184^®^) with melting glue (MG) based on EVA, including carbon black as filler in the ratio S77%/MG15%/CB8%. The composite generated internal Joule heating with a resistance of 3.5 ohm/cm by connecting the material to a circuit energized with 31 V. The material reached 150 °C, which was enough energy to melt the EVA polymer (T_m_ 75 °C) and promote its redistribution into the damaged region for healing [106] (Figure 12).

Sundaresan et al. developed a composite by blending a commercially available ionic polymer (Surlyn 8940) with carbon fiber. Surlyn films were made by compression molding by using polyimide films to prevent sticking of Surlyn films to the hot plates. Then films comprising Surlyn/carbon fiber/Surlyn sheets were compression molded generating the self-healing composite material. The fiber network of carbon was evenly distributed in the composite. The latter allowed generating temperature of 41 °C, 71 °C and 209 ° C by supplying voltages of 2V, 3V and 5V, corresponding to electrical currents of 0.571 A, 0.857A and 1.429 A, respectively. The self-healing process on damage regions was achieved after applying 4 V at about 2–3 W of power. In around 60 s, the composite reached the melting point of Surlyn, 95 °C, that allowed the polymer flowing and distributing in the damaged regions (cracks), recovering the original structure with 90% of efficiency [107].

Yang et al. reported the creation of a fiberglass-reinforced EVA composite mixed with -COOH-functionalized MWCNT as conductive filler. MWCNTs were dispersed in a surfactant solution where the fiberglass was submerged four times with drying steps in between. The laminate composite is manufactured by pressing the mix of EVA/fiberglass/MWCNTs at 170 °C. The MWCNTs provided the percolative conductive network and improved the mechanical performance of the composite due to the interfacial anchoring of MWCNTs between the fiberglass and the EVA matrix. Fatigue tests induced delamination that changed the electrical resistance of the material allowing the detection of the damage. To achieve self-healing, the material was subjected to a power of 0.18 W for 3 min generating temperatures above 75 °C, allowing EVA melting and flowing into cracks for damage recovery with an efficiency of about 88% [108].

**Figure 13:**
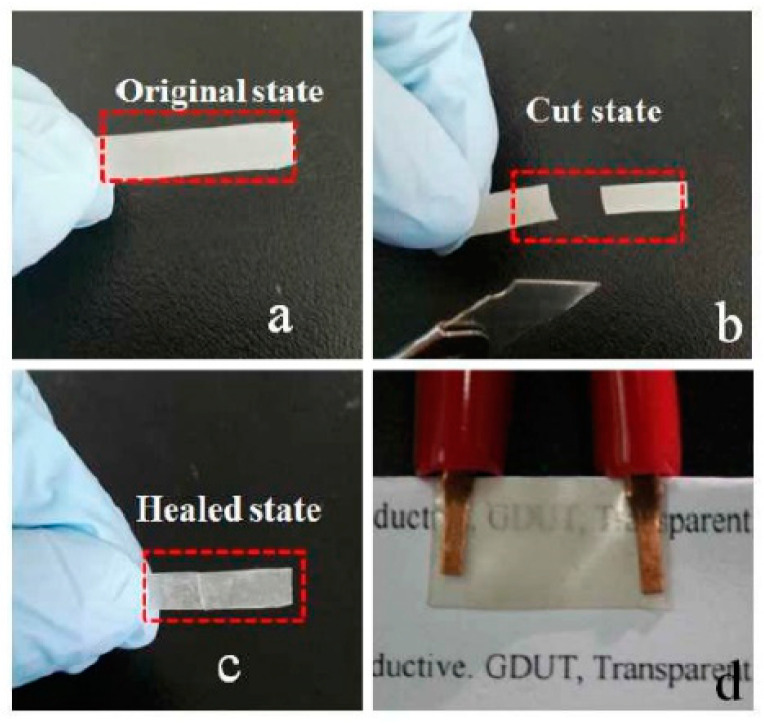
Illustration of healing process in epoxy/PCL blend mixed with silver nanowires: (**a**) original state, (**b**) after inducing cut; (**c**) after inducing electrical self-healing. (**d**) The composite turns transparent after healing in an oven [109]. Reproduced with permission from Luo, H; Pigment & Resin Technology; published by Esmerald Publishing, 2018.

Luo et al. designed an elastic epoxy/PCL blend mixed with silver nanowires (AgNWs) as conductive filler via dip coating [109]. The epoxy resin provided the shape memory effect to the material, while the PCL worked as a fusion agent for healing. The material was hot-compressed to achieve a percolating network of AgNWs conferring internal Joule heating when the material was subjected to electricity. It was was found that 50 wt.% of PCL provided the optimal concentration for the self-healing process. The composite displayed a resistivity of 4.1 ohm/mm and achieved a temperature of 105 °C after being connected to an electric circuit of 3 V and an electrical current of 0.008 A. After 10 s, the fusion and welding of the PCL allowed the self-healing and shape memory effect of the material to be obtained [109] (Figure 13).

**Figure 14:**
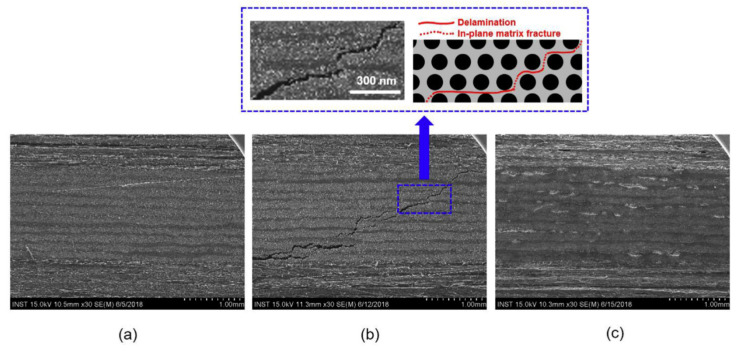
Cross sectional SEM images of carbon fiber reinforced polypropylene (CFPP) mixed with CNTs: (**a**) initial, (**b**) damaged, and (**c**) self-healed samples [110]. Reproduced with permission from Joo, S.J; Composites Science and Technology; published by Elsevier, 2018.

Joo et al. produced a composite based on unidirectional carbon fiber-reinforced polypropylene (CFPP) mixed with CNTs. The CNTs were dispersed in ethanol and spray coated on CFPPs, obtaining a laminated composite containing 1 wt.% CNTs. The composite displayed a resistivity of 19.44 ohm/mm, and after stimulating an electric current of 1.3 A for 30 min, the material generated a temperature of 181 °C. The temperature reached melt polypropylene, thus allowing the polymer to flow for self-healing on cracks with up to 96.83% efficiency [110] (Figure 14).

Chen et al. reported a self-healing composite by Joule effect produced by the coating of paraffin with PET films containing AgNWs. The sheet resistance of the composite was 88.6 ohm/sq and generated 75 °C after being connected to an electric circuit providing 12 V. The induced internal Joule heating generated enough energy to melt the paraffin and induce the crack healing effects [111].

Jimenez et al. generated a composite by mixing bisphenol A diglycidyl ether (DGEBA) and PCL at different ratios. The PCL and epoxy monomer were blended at 80 °C, adding MWCNTs from 0.05 to 0.2 wt.% as conductive filler. After that, 4,4 diaminophenol sulfone (DDS) was also added (curing agent) to reduce the viscosity of the mix at 180 °C. The mixture was poured into molds for curing at 210 °C over 3 h. After connecting the material to a circuit providing 145 V, the composite formulated with 20 wt.% of PCL and 0.02 wt.% of MWCNTs achieved a temperature of 100 °C by the Joule effect, thus melting the PCL, which flowed into the crack for self-healing [112].

**Figure 15:**
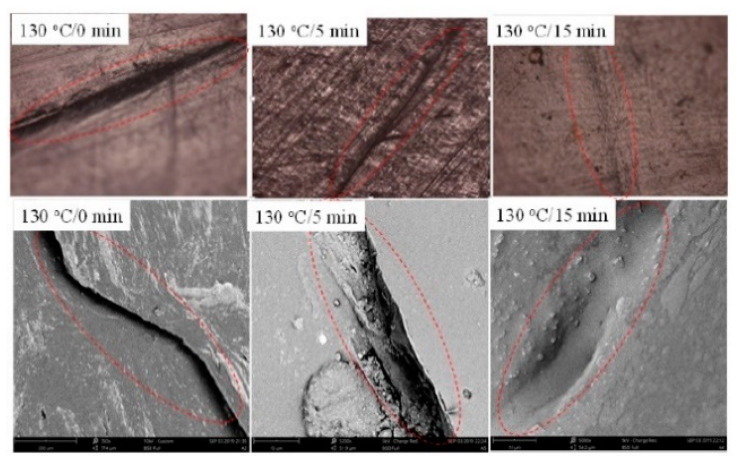
Optical and SEM images of scratch sample healed at 130 °C for deferent time using electricity [113]. Reproduced with permission from Wang, K; Nanomaterials; published by MDPI, 2020.

Wang et al. blended a thermoplastic polyurethane with 5 wt.% of graphene sheets to produce films by solvent casting. The film displayed a resistivity of 9.2 ohm/mm, and after applying a potential difference of 15 V, the material reached a temperature of 130 °C. The latter allowed the melting of the polyurethane, which flowed into cracks to achieve self-healing by the Joule effect [113] (Figure 15).

## 5. Extrinsic Self-Healing Materials by Joule Effect

Extrinsic self-healing materials use curative agents confined inside capsules and vascular systems embedded in polymer matrices. In general, curative agents are selected according to their chemical resistance to degradation and self-polymerization. They must possess a low freezing point and viscosity, low vapor pressure, and once released for healing, they must display high reactivity [114,115]. The main drawback of these systems is that recovery is limited to only a few times for areas of material due to the exhaustion of the curing agent [9,14,114,115].

### Covalent Bond

Kirkby et al. produced a self-healing composite displaying the shape memory effect (SME) by combining an epoxy resin (EPON 828, cured with diethylenetriamine (DETA)) as matrix with Ni:Ti:Cu alloy wires as conductive filler. The SME was achieved by thermal cycling. System contraction/expansion was observed due to solid–solid phase changes from austenite to martensite and vice versa. After applying tension to the wires, the healing in the composite was induced by adding 5 wt.% with Grubbs catalyst encapsulated in wax, and manually supplied into the crack. By applying an electrical current of 0.5 A for 10 min, the wire generated a temperature of 80 °C, thus triggering the polymerization of DCPD. At this temperature, the ring opening metathesis polymerization reaction (ROMP) was induced forming poly DCPD, achieving the self-healing of the material with a healing efficiency of 98% [116].

**Figure 16:**
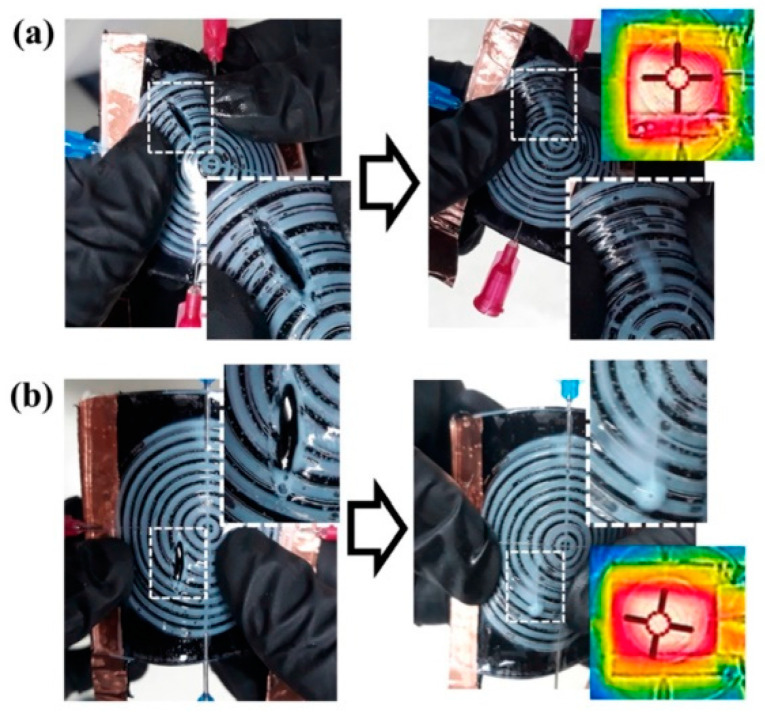
Self-healing of different cracks oriented at (**a**) 45° and (**b**) 90° [117]. Reproduced with permission from Kim, H; Polymer; published by Elsevier, 2019.

Kim et al. designed a system composed of an extrinsic self-healing material that consisted of a polydimethylsiloxane (PDMS) matrix containing dispersed CNTs as filler for external Joule heating. The authors produced two vascular systems, one containing the curing agent, and the other containing the DMS resin, both placed on the PDMS film. This system is autonomous by itself, since it can produce the self-healing at room temperature, although it takes 24 h to repair the cracks. The Joule effect was used to increase the viscosity of the curing agents, so that these components could flow to the cracks. The application of a potential difference of 9 V generated an electric current of 1.06 A, which increased the temperature to 126.8 °C, thus reducing the curing time from 24 h to only 10 min. The material recovered its mechanical properties after damage up to 90%. The authors also tested these healing properties at low temperatures (1 °C), opening possibilities for the use of these materials in severe climates [117] (Figure 16).

We can see from Table 1 that the type of self-healing most used for self-healing materials by the Joule effect are those possessing an intrinsic nature. The vast majority are supramolecular interaction mechanisms followed by Diels-Alder mechanisms. It can also be observed that the most used filler is MWCNTs and the entire family of conductive fillers of the carbonaceous family, such as SWCNT, graphene and carbon black. There are also metal fillers, such as AgNWs, which open the way for transparent applications. Regarding self-healing efficiencies, we can see that the most efficient mechanisms are those based on intrinsic Diels-Alder reversible cycloaddition. Moreover, the Au-S link shows an efficiency of 90% after the second cycle, and the alkoxyamines reach up to 81.4%. It can also be observed that supramolecular systems present good self-healing yields with 70% for the lowest and 97% for the highest efficiency. The lower performance is presented by BIIR ionic systems with imidazole, which lose from 45 to 60% of their original elongation.

## 6. Discussion

Self-repair in polymer nanocomposites can be achieved by different chemical and physical approaches as discussed in the previous sections. To trigger the healing properties by the Joule effect, a nanocomposite must be both thermally and electrically conductive and must exhibit thermally self-healing properties. To achieve this combination of properties, polymer matrices are combined with conductive fillers, providing synergistic effects of both components such as percolative pathways that help to carry electrical and thermal energy for self-healing processes generated from the Joule effect. Intrinsic systems rely mainly on the matrix ability for self-healing, which in turn depends on the molecular weight of the polymer, its viscoelastic behavior, and the activation/reactivity of functional groups at crack interfaces. Particularly, the interfacial interaction between matrix and filler appears to be a crucial factor determining the effectiveness of healing activation of intrinsic self-healing systems. Anchoring the polymer to the filler leads to better mechanical performance of the system. However, it might influence the free volume and flow of polymer chains for healing mechanisms. The entanglement, wetting and physical/chemical interaction of polymer chains plays crucial roles in crack healing, which will in turn provide optimal recovery of mechanical performance and healing efficiency. The main task is to find the ideal electrical conditions for a correct and efficient self-healing, considering the optimal ratio between the components and reactivity without sacrificing mechanical performance.

Among many fillers, carbonaceous and metallic fibers and nanostructures are the most used ones in combination with polymer matrices [38,87]. These fillers provide thermal and electrical conductivity to the composite. Through these, electricity can be supplied to the systems as external energy source to generate heat by Joule effect. This energy can activate properties such as shape memory [88,116] and self-healing in functionalized composites [53,94]. To generate internal Joule heating through nanofillers such as graphene, carbon nanotubes, gold nanoparticles, carbon black, silver and copper nanowires, the fillers must be properly dispersed and stabilized to fulfill percolative pathways. The latter comprises an infinite network that presents random paths throughout the material generated by a tunneled electron transport networks [79]. Effective dispersion and stabilization prevent the aggregation of the filler for optimal energy transport. This is fulfilled by the functionalization of the filler surface (oxidation/reduction), so that it displays better interfacial interaction with polymer matrices. The problem of excessive functionalization, particularly for graphitic carbonaceous materials, is that the hybridization of carbons changes from planar (sp^2^) to tetrahedral (sp^3^), which causes the decrease in electrical conduction and mechanical performance of the entire system. Additionally, excessive coating on the fillers by the matrix may hinder filler contacts, thus decreasing the percolative filler network. As a result, the conduction is not achieved prompting to increase energy input and filler concentration for sufficient percolative pathways. The minimum filler concentration needed to form a percolative network must be pinpointed to avoid excessive filler concentration before reaching the composite failure due to the lack of effective interfacial interaction between matrix and filler [79].

For the design of these materials, it is also necessary to consider the effect of the positive and negative temperature coefficient of expansion that can occur in the material. The latter might considerably affect the conductivity of the systems during heating procedures. In particular, the cooling cycles might break the percolative network of nanofillers hindering the healing ability by Joule effect. Therefore, the amorphous and crystalline degrees of a polymer/blend matrix are particularly important to control the effective interfacial interaction between matrix and filler. Nanofillers showing electrical/thermal conductivity in the polymeric matrix must provide heat distribution uniformly throughout the material, so that the energy is used optimally. Finally, it is necessary for the filler network to be correctly formed so that during heating-cooling cycles, the percolating network will not be lost, and thus the self-healing ability, recyclable and reprocessability of the polymer matrix will not be hampered [118,119].

## 7. Conclusions

Self-healing materials activated by the heat produced by the Joule effect are a family of materials that have the characteristics of both electrical/thermal conduction and self-recovery by different chemical mechanisms. External and internal Joule heating composites systems are increasingly being reported in the scientific literature. Works based on intrinsic self-healing materials led by Diels-Alder cycloaddition chemistry and supramolecular interactions are the most frequently reported ones. A few examples of extrinsic self-healing nanocomposite materials have also been reported, opening great opportunities for new investigations.

The main characteristics of the reported systems is that the composite materials must be conductive and possess a type of self-healing mechanism activated by temperature. The first approaches for achieving this kind of technology were composite materials based on polymers and conductive fibers with optimal results regarding self-healing ability. However, problems such as fiber breaks hinder the conduction of the material. As a solution, nanofillers give a great leap forward for this technology, since the conduction of the material becomes an intrinsic part of it. As damage occurs, the healing system behavior is intrinsic in nature, making it possible to achieve around 100% functionality after damage/healing procedures.

Although the mechanical stability of intrinsic self-healing composites is compromised when acting at the softening temperatures of the material, nevertheless, fillers help to support the dimensional stability of the materials by interfacial interactions (chemical/physical) that reinforce the whole system, resulting in high-temperature melting points.

With the advances at the nanoscopic levels, microcracks can be repaired very effectively. However, larger cracks need much research to generate highly efficient nanocomposites. For instance, shape memory assisted self-healing polymer composites are a great approach that works by reducing macro-sized cracks to microcracks as a result of the shape memory effect provided by entropic energy stored and junction points (physical and chemical reversible crosslinking) [118]. These types of materials display low softening temperature, making them flexible for application as smart materials.

As a future perspective, it has been well observed that self-healing composites have the advantage of being produced by a great quantity of bulk materials at industrial scale. Among them, epoxy monomers, EVA and PCL, and conductive nano/fillers such as carbon fibers, graphitic particles, and metal wires are currently commercially available. Multifunctional polymers with reactive chemical groups that can act as good stabilizers of nanofillers, while at the same time showing self-healing ability, are also a reality nowadays. However, much more effort must be devoted towards their production at industrial scale for real-world applications. Specifically, it is necessary to consider the effect of the positive and negative temperature coefficients that can occur in the material during healing procedures by the Joule effect. The latter is related to increasing/decreasing the temperature and raising/lowering the conductivity of the nanocomposite. It is indeed crucial, since heating and cooling cycles might break the percolative filler network rendering internal stresses to the material which has a direct correlation with the amorphous and crystalline degree of the matrix. Additionally, the thermal conductivity of the polymeric matrix is an important factor to consider, because the heat distribution must be uniform throughout the material, so that the energy used for healing will be optimal.

Finally, the Joule effect has attractive characteristics for developing self-healing smart materials, since the increase of temperature at localized regions in material failures is useful for early detection, thus avoiding critical damage. At the same time, the electrical stimulus as trigger for self-healing effects is a simple and efficient economical way to repair materials in service, thus avoiding material replacement, maintenance and early disposal.

## Figures and Tables

**Table 1:** Summary of self-healing materials by Joule effect.

Matrix.	Filler	Mechanism		Volt (V)	Current (A)	T (°C)	Healing Efficiency (%)	Ref.
		Intrinsic	Extrinsic					
Mendomer/epoxy	Carbon fiber	Diels-Alder	X	X	2	<100	X	[38]
Mendomer 401	Carbon fiber	Diels-Alder	X	X	0.5	150	92.3	[87]
Tetrafuran/bismaleimide	Carbon fiber	Diels-Alder	X	X	1.2	110	87.5	[88]
Polyurethane	MWCNT	Diels-Alder	X	25	X	110	X	[89]
Polyurethane	AgNW	Diels-Alder	X	X	X	120	X	[90]
Polyketone	MWCNT	Diels-Alder	X	25	X	50–60	X	[79]
Polyurethane	CNT	Diels-Alder	X	20	X	106	98	[53]
Polyketone	MWCNT	Diels-Alder	X	25–50	X	120–150	X	[91]
Polyurethane modified with alkoxyamine	MWCNT	Alkoxyamine	X	X	X	110–200	81.4	[94]
Nanobelt Au/BACA	NP Au	Au-S link	X	X	0.005	X	90	[97]
BIIR modified with imidazole	CNT	Ionic	X	15	X	100	40.5	[101]
BIIR modified with imidazole	CNT	Ionic	X	38	X	110–200	54.2	[102]
BIIR	Pet/CuNF	Ionic	X	1.3	1.8	100	X	[62]
BIIR	MWCNT	Ionic	X	28.5	X	150	X	[103]
Silicone/EVA	NiTi	Supramolecular	X	6	X	X	80	[105]
Silicone/EVA	Black carbon	Supramolecular	X	31	X	X	X	[106]
Surlyn 8940	Carbon fiber	Supramolecular	X	5	X	95	80	[107]
Eva/Glass fiber	MWCNT	Supramolecular	X	X	X	X	80.5	[108]
Epoxy/PCL	AgNW	Supramolecular	X	3	0.008	105	70	[109]
CFRPP	CNT	Supramolecular	X	X	1.3	181	96.8	[110]
PET/Paraffin	AgNW	Supramolecular	X	12	X	75	X	[111]
Epoxy/PCL	MWCNT	Supramolecular	X	145	X	100	X	[112]
Thermoplastic polyurethane	Graphene	Supramolecular	X	15	X	130	X	[113]
EPON 828	Ni:Ti:Cu wire	X	Covalent bond	X	0.5	80	50	[116]
PDMS	CNT	X	Covalent bond	X	1.06	126.8	110	[117]

X Information not available.

## References

[1] Chan Y.L., Ngan A.H.W., King N.M. (2012). Nanomechanical Characterization of Mineralized Tissues in the Oral Cavity. Emerging Nanotechnologies in Dentistry.

[2] Döhler D., Michael P., Binder W. (2013). Principles of Self-Healing Polymers. Self-Healing Polymers.

[3] Wool R.P. (2008). Self-healing materials: A review. Soft Matter.

[4] Thakur V.K., Kessler M.R. (2015). Self-healing polymer nanocomposite materials: A review. Polymer.

[5] Urdl K., Kandelbauer A., Kern W., Müller U., Thebault M., Zikulnig-Rusch E. (2017). Self-healing of densely crosslinked thermoset polymers—A critical review. Prog. Org. Coat..

[6] Hu J. (2013). Introduction to shape memory polymers. Advances in Shape Memory Polymers.

[7] Ahner J., Bode S., Micheel M., Dietzek B., Hager M.D. (2015). Self-Healing Functional Polymeric Materials. Advances in Polymer Science.

[8] Kang J., Tok J.B.-H., Bao Z. (2019). Self-healing soft electronics. Nat. Electron..

[9] Mobaraki M., Ghaffari M., Mozafari M. (2020). Basics of self-healing composite materials. Self-Healing Composite Materials.

[10] Paul D.R., Robeson L.M. (2008). Polymer nanotechnology: Nanocomposites. Polymer.

[11] Balazs A.C., Emrick T., Russell T.P. (2006). Nanoparticle Polymer Composites: Where Two Small Worlds Meet. Science.

[12] Wu D.Y., Meure S., Solomon D. (2008). Self-healing polymeric materials: A review of recent developments. Prog. Polym. Sci..

[13] Yan X., Wang F., Zheng B., Huang F. (2012). Stimuli-responsive supramolecular polymeric materials. Chem. Soc. Rev..

[14] Kuhl N., Bode S., Hager M.D., Schubert U.S. (2015). Self-Healing Polymers Based on Reversible Covalent Bonds. Advances in Polymer Science.

[15] Leng J., Lan X., Liu Y., Du S. (2010). Multifunctional Polymeric Smart Materials. Multifunctional Polymer Nanocomposites.

[16] Zhang Z.P., Rong M.Z., Zhang M.Q. (2018). Polymer engineering based on reversible covalent chemistry: A promising innovative pathway towards new materials and new functionalities. Prog. Polym. Sci..

[17] Chandra A.K., Kumar N.R. (2017). Polymer Nanocomposites for Automobile Engineering Applications. Properties and Applications of Polymer Nanocomposites.

[18] Gowri S., Almeida L., Amorim T., Carneiro N., Souto A.P., Esteves M.F. (2010). Polymer Nanocomposites for Multifunctional Finishing of Textiles—A Review. Text. Res. J..

[19] Tanaka T. (2005). Dielectric nanocomposites with insulating properties. IEEE Trans. Dielectr. Electr. Insul..

[20] Hopewell J., Dvorak R., Kosior E. (2009). Plastics recycling: Challenges and opportunities. Philos. Trans. R. Soc. B Biol. Sci..

[21] Schneiderman D.K., Hillmyer M.A. (2017). 50th Anniversary Perspective: There Is a Great Future in Sustainable Polymers. Macromolecules.

[22] Toncelli C., De Reus D., Broekhuis A., Picchioni F. (2011). Thermoreversibility in Polymeric Systems. Self-Healing at the Nanoscale.

[23] Kim J.-W., Sauti G., Siochi E.J., Smith J.G., Wincheski R.A., Cano R.J., Connell J.W., Wise K.E. (2014). Toward High Performance Thermoset/Carbon Nanotube Sheet Nanocomposites via Resistive Heating Assisted Infiltration and Cure. ACS Appl. Mater. Interfaces.

[24] Lee J., Stein I.Y., Kessler S.S., Wardle B.L. (2015). Aligned Carbon Nanotube Film Enables Thermally Induced State Transformations in Layered Polymeric Materials. ACS Appl. Mater. Interfaces.

[25] Toohey K.S., Sottos N.R., Lewis J.A., Moore J.S., White S.R. (2007). Self-healing materials with microvascular networks. Nat. Mater..

[26] Blaiszik B.J., Sottos N.R., White S.R. (2008). Nanocapsules for self-healing materials. Compos. Sci. Technol..

[27] Yuan Y.C., Yin T., Rong M.Z., Zhang M.Q. (2008). Self healing in polymers and polymer composites. Concepts, realization and outlook: A review. Express Polym. Lett..

[28] Utrera-Barrios S., Verdejo R., López-Manchado M., Santana M.H. (2020). Evolution of self-healing elastomers, from extrinsic to combined intrinsic mechanisms: A review. Mater. Horiz..

[29] Bose R.K., Picchioni F., Muljana H. (2019). Thermoreversible Polymeric Nanocomposites. Nanocomposites—Recent Evolutions.

[30] Taylor D.L., Panhuis M.I.H. (2016). Self-Healing Hydrogels. Adv. Mater..

[31] Gallego J., Del Val M.A., Contreras V., Páez A. (2013). Heating asphalt mixtures with microwaves to promote self-healing. Constr. Build. Mater..

[32] (2003). Microwave synthesis: Chemistry at the speed of light. Choice Rev. Online.

[33] Huang L., Li J., Yuan W., Liu X., Li Z., Zheng Y., Liang Y., Zhu S., Cui Z., Yang X. (2020). Near-infrared light controlled fast self-healing protective coating on magnesium alloy. Corros. Sci..

[34] Cheng Y., Ren K., Huang C., Wei J. (2019). Self-healing graphene oxide-based nanocomposite hydrogels serve as near-infrared light-driven valves. Sens. Actuators B Chem..

[35] Hohlbein N., Shaaban A., Schmidt A.M. (2015). Remote-controlled activation of self-healing behavior in magneto-responsive ionomeric composites. Polymer.

[36] Duenas T., Enke A., Chai K., Castellucci M., Sundaresan V.B., Wudl F., Murphy E.B., Mal A., Alexandar J.R., Corder A. (2010). Smart Self-Healing Material Systems Using Inductive and Resistive Heating. ACS Symposium Series.

[37] Sastry S.K., Barach J.T. (2000). Ohmic and Inductive Heating. J. Food Sci..

[38] Park J.S., Takahashi K., Guo Z., Wang Y., Bolanos E., Hamann-Schaffner C., Murphy E., Wudl F., Hahn H.T. (2008). Towards Development of a Self-Healing Composite using a Mendable Polymer and Resistive Heating. J. Compos. Mater..

[39] Zhang Y., Broekhuis A.A., Picchioni F. (2009). Thermally Self-Healing Polymeric Materials: The Next Step to Recycling Thermoset Polymers?. Macromolecules.

[40] Korhonen J., Honkasalo A., Seppälä J. (2018). Circular Economy: The Concept and its Limitations. Ecol. Econ..

[41] Hia I.L., Vahedi V., Pasbakhsh P. (2016). Self-Healing Polymer Composites: Prospects, Challenges, and Applications. Polym. Rev..

[42] Drude P. (1900). Zur Elektronentheorie der Metalle. Ann. Phys..

[43] Kwok N., Hahn H.T. (2007). Resistance Heating for Self-healing Composites. J. Compos. Mater..

[44] Otten R.H.J., Van Der Schoot P. (2009). Continuum Percolation of Polydisperse Nanofillers. Phys. Rev. Lett..

[45] Otten R.H.J., Van Der Schoot P. (2011). Connectivity percolation of polydisperse anisotropic nanofillers. J. Chem. Phys..

[46] Tjong S.C. (2014). Polymer composites with graphene nanofillers: Electrical properties and applications. J. Nanosci. Nanotechnol..

[47] Li Y., Huang X., Zeng L., Li R., Tian H., Fu X., Wang Y., Zhong W.-H. (2019). A review of the electrical and mechanical properties of carbon nanofiller-reinforced polymer composites. J. Mater. Sci..

[48] Sakagami T., Ogura K. (1992). A New Flaw Inspection Technique Based on Infrared Thermal Images under Joule Effect Heating. Trans. Jpn. Soc. Mech. Eng. Ser. A.

[49] Liu T.J.-C. (2014). Joule heating behaviors around through crack emanating from circular hole under electric load. Eng. Fract. Mech..

[50] Cai G.X., Yuan F.G. (1999). Electric Current-Induced Stresses at the Crack Tip in Conductors. Int. J. Fract..

[51] Yu J., Zhang H., Deng D., Hao S., Iqbal A. (2014). Numerical calculation and experimental research on crack arrest by detour effect and joule heating of high pulsed current in remanufacturing. Chin. J. Mech. Eng..

[52] Oswald-Tranta B. (2018). Induction Thermography for Surface Crack Detection and Depth Determination. Appl. Sci..

[53] Pu W., Fu D., Wang Z., Gan X., Lu X., Yang L., Xia H. (2018). Realizing Crack Diagnosing and Self-Healing by Electricity with a Dynamic Crosslinked Flexible Polyurethane Composite. Adv. Sci..

[54] Huang J.-C. (2002). Carbon black filled conducting polymers and polymer blends. Adv. Polym. Technol..

[55] Mittal G., Dhand V., Rhee K.Y., Park S.-J., Lee W.R. (2015). A review on carbon nanotubes and graphene as fillers in reinforced polymer nanocomposites. J. Ind. Eng. Chem..

[56] Atif R., Shyha I., Inam F. (2016). Mechanical, Thermal, and Electrical Properties of Graphene-Epoxy Nanocomposites—A Review. Polymers.

[57] Imtiaz S., Siddiq M., Kausar A., Muntha S.T., Ambreen J., Bibi I. (2018). A Review Featuring Fabrication, Properties and Applications of Carbon Nanotubes (CNTs) Reinforced Polymer and Epoxy Nanocomposites. Chin. J. Polym. Sci..

[58] Srivastava S., Mishra Y. (2018). Nanocarbon Reinforced Rubber Nanocomposites: Detailed Insights about Mechanical, Dynamical Mechanical Properties, Payne, and Mullin Effects. Nanomaterials.

[59] Mamidi N., Delgadillo R.V., Ortiz A.G., Barrera E. (2020). Carbon Nano-Onions Reinforced Multilayered Thin Film System for Stimuli-Responsive Drug Release. Pharmaceutics.

[60] Nam S., Kim Y., Shim H.-S., Kim J., Kim W. (2011). Copper nanofiber-networked cobalt oxide composites for high performance Li-ion batteries. Nanoscale Res. Lett..

[61] Abbasi N.M., Yu H., Wang L., Abdin Z.U., Amer W.A., Akram M., Khalid H., Chen Y., Saleem M., Sun R. (2015). Preparation of silver nanowires and their application in conducting polymer nanocomposites. Mater. Chem. Phys..

[62] Lee M.W., Jo H.S., Yoon S.S., Yarin A.L. (2017). Thermally driven self-healing using copper nanofiber heater. Appl. Phys. Lett..

[63] Shah K.W., Xiong T. (2019). Multifunctional Metallic Nanowires in Advanced Building Applications. Materials.

[64] Wang L., Aslani F. (2019). A review on material design, performance, and practical application of electrically conductive cementitious composites. Constr. Build. Mater..

[65] Papanastasiou D.T., Schultheiss A., Muñoz-Rojas D., Celle C., Carella A., Simonato J., Bellet D. (2020). Transparent Heaters: A Review. Adv. Funct. Mater..

[66] Liu H., Li Q., Zhang S., Yin R., Liu X., He Y., Dai K., Shan C., Guo J., Liu C. (2018). Electrically conductive polymer composites for smart flexible strain sensors: A critical review. J. Mater. Chem. C.

[67] Kaur G., Adhikari R., Cass P., Bown M., Gunatillake P. (2015). Electrically conductive polymers and composites for biomedical applications. RSC Adv..

[68] Aradhana R., Mohanty S., Nayak S.K. (2020). A review on epoxy-based electrically conductive adhesives. Int. J. Adhes. Adhes..

[69] Marsden A.J., Papageorgiou D.G., Vallés C., Liscio A., Palermo V., Bissett M.A., Young R.J., Kinloch I.A. (2018). Electrical percolation in graphene–polymer composites. 2D Mater..

[70] Park M., Park J., Jeong U. (2014). Design of conductive composite elastomers for stretchable electronics. Nano Today.

[71] Deng H., Lin L., Ji M., Zhang S., Yang M., Fu Q. (2014). Progress on the morphological control of conductive network in conductive polymer composites and the use as electroactive multifunctional materials. Prog. Polym. Sci..

[72] Chen H., Ginzburg V.V., Yang J., Yang Y., Liu W., Huang Y., Du L., Chen B. (2016). Thermal conductivity of polymer-based composites: Fundamentals and applications. Prog. Polym. Sci..

[73] Li A., Zhang C., Zhang Y.F. (2017). Thermal Conductivity of Graphene-Polymer Composites: Mechanisms, Properties, and Applications. Polymers.

[74] Huang X., Zhi C., Lin Y., Bao H., Wu G., Jiang P., Mai Y.-W. (2020). Thermal conductivity of graphene-based polymer nanocomposites. Mater. Sci. Eng. R Rep..

[75] Kloxin C.J., Scott T.F., Adzima B.J., Bowman C.N. (2010). Covalent Adaptable Networks (CANs): A Unique Paradigm in Cross-Linked Polymers. Macromolecules.

[76] Kloxin C.J., Bowman C.N. (2013). Covalent adaptable networks: Smart, reconfigurable and responsive network systems. Chem. Soc. Rev..

[77] Munirasu S., Albuerne J., Boschetti-De-Fierro A., Abetz V. (2010). Functionalization of Carbon Materials using the Diels-Alder Reaction. Macromol. Rapid Commun..

[78] Polgar L., Criscitiello F., Van Essen M., Araya-Hermosilla R., Migliore N., Lenti M., Raffa P., Picchioni F., Pucci A. (2018). Thermoreversibly Cross-Linked EPM Rubber Nanocomposites with Carbon Nanotubes. Nanomaterials.

[79] Araya-Hermosilla R., Pucci A., Raffa P., Santosa D., Daems N., Gengler R.Y.N., Rudolf P., Moreno-Villoslada I., Picchioni F. (2018). Electrically-Responsive Reversible Polyketone/MWCNT Network through Diels-Alder Chemistry. Polymers.

[80] Yang Y., Ding X., Urban M.W. (2015). Chemical and physical aspects of self-healing materials. Prog. Polym. Sci..

[81] Liu Y.-L., Chuo T.-W. (2013). Self-healing polymers based on thermally reversible Diels–Alder chemistry. Polym. Chem..

[82] Chang C.-M., Liu Y.-L. (2009). Functionalization of multi-walled carbon nanotubes with furan and maleimide compounds through Diels–Alder cycloaddition. Carbon.

[83] Gandini A. (2013). The furan/maleimide Diels–Alder reaction: A versatile click–unclick tool in macromolecular synthesis. Prog. Polym. Sci..

[84] Bergman S.D., Wudl F. (2008). Mendable polymers. J. Mater. Chem..

[85] Syrett J.A., Becer C.R., Haddleton D.M. (2010). Self-healing and self-mendable polymers. Polym. Chem..

[86] Murphy E.B., Wudl F. (2010). The world of smart healable materials. Prog. Polym. Sci..

[87] Park J.S., Kim H.S., Hahn H.T. (2009). Healing behavior of a matrix crack on a carbon fiber/mendomer composite. Compos. Sci. Technol..

[88] Park J.S., Darlington T., Starr A.F., Takahashi K., Riendeau J., Hahn H.T. (2010). Multiple healing effect of thermally activated self-healing composites based on Diels–Alder reaction. Compos. Sci. Technol..

[89] Willocq B., Bose R.K., Khelifa F., Garcia S.J., Dubois P., Raquez J.-M. (2016). Healing by the Joule effect of electrically conductive poly(ester-urethane)/carbon nanotube nanocomposites. J. Mater. Chem. A.

[90] Tiwari N., Ankit A., Rajput M., Kulkarni M.R., John R.A., Mathews N. (2017). Healable and flexible transparent heaters. Nanoscale.

[91] Lima G.R.M., Orozco F., Picchioni F., Moreno-Villoslada I., Pucci A., Bose R.K., Araya-Hermosilla R., Lima M., Villoslada M. (2019). Electrically Self-Healing Thermoset MWCNTs Composites Based on Diels-Alder and Hydrogen Bonds. Polymers.

[92] Moad G., Rizzardo E. (1995). Alkoxyamine-Initiated Living Radical Polymerization: Factors Affecting Alkoxyamine Homolysis Rates. Macromolecules.

[93] Yuan C., Zhang M.Q., Rong M.Z. (2014). Application of alkoxyamine in self-healing of epoxy. J. Mater. Chem. A.

[94] Fan L.F., Rong M.Z., Zhang M.Q., Chen X.D. (2018). Repeated Intrinsic Self-Healing of Wider Cracks in Polymer via Dynamic Reversible Covalent Bonding Molecularly Combined with a Two-Way Shape Memory Effect. ACS Appl. Mater. Interfaces.

[95] Casuso P., Odriozola I., Vicente A.P.-S., Loinaz I., Cabañero G., Grande H.-J., Dupin D. (2015). Injectable and Self-Healing Dynamic Hydrogels Based on Metal(I)-Thiolate/Disulfide Exchange as Biomaterials with Tunable Mechanical Properties. Biomacromolecules.

[96] Qin H., Zhang T., Li H.-N., Cong H.-P., Antonietti M., Yu S.-H. (2017). Dynamic Au-Thiolate Interaction Induced Rapid Self-Healing Nanocomposite Hydrogels with Remarkable Mechanical Behaviors. Chem.

[97] Wu B.S., Ye Y.C., Li Z., Liu Z.Y., Pei Y.Y., Chen C.R., Qin H.L., Liu H.H. (2019). Fabrication and Property of Electric-Induced Self-Healing Nanocomposite Hydrogels. Acta Polym. Sin..

[98] Das A., Sallat A., Böhme F., Suckow M., Basu D., Wiessner S., Stöckelhuber K.W., Voit B., Heinrich G. (2015). Ionic Modification Turns Commercial Rubber into a Self-Healing Material. ACS Appl. Mater. Interfaces.

[99] Xu C., Cao L., Lin B., Liang X., Chen Y. (2016). Design of Self-Healing Supramolecular Rubbers by Introducing Ionic Cross-Links into Natural Rubber via a Controlled Vulcanization. ACS Appl. Mater. Interfaces.

[100] Sallat A., Das A., Schaber J., Scheler U., Bhagavatheswaran E.S., Stöckelhuber K.W., Heinrich G., Voit B., Böhme F. (2018). Viscoelastic and self-healing behavior of silica filled ionically modified poly(isobutylene-co-isoprene) rubber. RSC Adv..

[101] Le H.H., Hait S., Das A., Wiessner S., Stoeckelhuber K.W., Boehme F., Reuter U., Naskar K., Heinrich G., Radusch H.-J. (2017). Self-healing properties of carbon nanotube filled natural rubber/bromobutyl rubber blends. Express Polym. Lett..

[102] Le H.H., Böhme F., Sallat A., Wießner S., Der Landwehr M.A., Reuter U., Stöckelhuber K.-W., Heinrich G., Radusch H.-J., Das A. (2017). Triggering the Self-Healing Properties of Modified Bromobutyl Rubber by Intrinsically Electrical Heating. Macromol. Mater. Eng..

[103] Kim H., Yarin A.L., Lee M.W. (2020). Self-healing corrosion protection film for marine environment. Compos. Part B Eng..

[104] Yang Y., Urban M.W. (2013). Self-healing polymeric materials. Chem. Soc. Rev..

[105] Wang C.C., Ding Z., Purnawali H., Huang W.M., Fan H., Sun L. (2012). Repeated Instant Self-healing Shape Memory Composites. J. Mater. Eng. Perform..

[106] Cui H.P., Song C.L., Huang W.M., Wang C.C., Zhao Y. (2013). Rubber-like electrically conductive polymeric materials with shape memory. Smart Mater. Struct..

[107] Sundaresan V.B., Morgan A., Castellucci M. (2013). Self-Healing of Ionomeric Polymers with Carbon Fibers from Medium-Velocity Impact and Resistive Heating. Smart Mater. Res..

[108] Yang B., Xuan F.-Z., Wang Z., Chen L., Lei H., Liang W., Xiang Y., Yang K. (2018). Multi-functional interface sensor with targeted IFSS enhancing, interface monitoring and self-healing of GF/EVA thermoplastic composites. Compos. Sci. Technol..

[109] Luo H., Zhou X., Xu Y., Wang H., Yao Y., Yi G., Hao Z. (2018). Multi-stimuli triggered self-healing of the conductive shape memory polymer composites. Pigment. Resin Technol..

[110] Joo S.-J., Yu M.-H., Kim W.S., Kim H.-S. (2018). Damage detection and self-healing of carbon fiber polypropylene (CFPP)/carbon nanotube (CNT) nano-composite via addressable conducting network. Compos. Sci. Technol..

[111] Chen C., Huang Z., Jiao Y., Shi L.-A., Zhang Y., Li J., Li C., Lv X., Wu S., Hu Y. (2019). In Situ Reversible Control between Sliding and Pinning for Diverse Liquids under Ultra-Low Voltage. ACS Nano.

[112] Jiménez-Suárez A., Martín-González J., Sánchez-Romate X.F., Prolongo S.G. (2020). Carbon nanotubes to enable autonomous and volumetric self-heating in epoxy/polycaprolactone blends. Compos. Sci. Technol..

[113] Wang K., Zhou Z., Zhang J., Tang J., Wu P., Wang Y., Zhao Y., Leng Y. (2020). Electrical and Thermal and Self-Healing Properties of Graphene-Thermopolyurethane Flexible Conductive Films. Nanomaterials.

[114] Billiet S., Hillewaere X.K.D., Teixeira R.F.A., Du Prez F.E. (2013). Chemistry of Crosslinking Processes for Self-Healing Polymers. Macromol. Rapid Commun..

[115] Zhu D.Y., Rong M.Z., Zhang M.Q. (2015). Self-healing polymeric materials based on microencapsulated healing agents: From design to preparation. Prog. Polym. Sci..

[116] Kirkby E.L., Rule J.D., Michaud V.J., Sottos N.R., White S.R., Månson J.-A.E. (2008). Embedded Shape-Memory Alloy Wires for Improved Performance of Self-Healing Polymers. Adv. Funct. Mater..

[117] Kim H., Yarin A.L., Lee M.W. (2019). Ultra-fast bull’s eye-like self-healing using CNT heater. Polymer.

[118] Hornat C.C., Urban M.W. (2020). Entropy and interfacial energy driven self-healable polymers. Nat. Commun..

[119] Aharony A. (2018). Introduction: Forest Fires, Fractal Oil Fields, and Diffusion. Introduction to Percolation Theory.

